# Genotype by Prenatal Environment Interaction for Postnatal Growth of Nelore Beef Cattle Raised under Tropical Grazing Conditions

**DOI:** 10.3390/ani13142321

**Published:** 2023-07-15

**Authors:** Mário L. Santana, Annaiza B. Bignardi, Rodrigo J. Pereira, Gerson A. Oliveira Junior, Anielly P. Freitas, Roberto Carvalheiro, Joanir P. Eler, José B. S. Ferraz, Joslaine N. S. G. Cyrillo, Maria E. Z. Mercadante

**Affiliations:** 1 Grupo de Melhoramento Animal de Mato Grosso (GMAT), Instituto de Ciências Agrárias e Tecnológicas, Universidade Federal de Rondonópolis (UFR), Rondonópolis 78735-901, Brazil; 2 Centre for Genetic Improvement of Livestock, Department of Animal Biosciences, University of Guelph, Guelph, ON N1G 2W1, Canada; 3 Centro de Pesquisa em Bovinos de Corte, Instituto de Zootecnia (IZ), Sertãozinho 14160-900, Brazil; 4 Faculdade de Ciências Agrárias e Veterinárias, Universidade Estadual Paulista (UNESP), Jaboticabal 14884-900, Brazil; 5 Grupo de Melhoramento Animal e Biotecnologia (GMAB), Departamento de Medicina Veterinária, FZEA, Universidade de São Paulo (USP), Pirassununga 13635-900, Brazil

**Keywords:** developmental programming, epigenetic, fetal programming, genotype by environment interaction, GWAS, Nellore, reaction norms, SNP, zebu

## Abstract

**Simple Summary:**

The prenatal environment can influence the postnatal performance of cattle. Especially in tropical regions, pregnant beef cows may experience nutritional restriction during gestation, which coincides with the season of poor quality and quantity of feed. Thus, it was verified that the offspring of cows subjected to a better gestation environment exhibited better productive and reproductive performances throughout their lives. In terms of genetic merit, it was found that the best animals in a restricted gestational environment are not necessarily the same in a favorable gestational environment. In other words, for each condition of the gestational environment, there are animals specifically suited to perform better. In addition, regions in the genome of these animals responsible for several traits of economic importance in cattle were identified. Thus, for a more efficient selection process, breeders must consider the effect of genotype by prenatal environment interaction and provide adequate management and nutrition care for pregnant cows.

**Abstract:**

The prenatal environment is recognized as crucial for the postnatal performance in cattle. In tropical regions, pregnant beef cows commonly experience nutritional restriction during the second half of the gestation period. Thus, the present study was designed to analyze the genotype by prenatal environment interaction (G × Epn) and to identify genomic regions associated with the level and response in growth and reproduction-related traits of beef cattle to changes in the prenatal environment. A reaction norm model was applied to data from two Nelore herds using the solutions of contemporary groups for birth weight as a descriptor variable of the gestational environment quality. A better gestational environment favored weights until weaning, scrotal circumference at yearling, and days to first calving of the offspring. The G × Epn was strong enough to result in heterogeneity of variance components and genetic parameters in addition to reranking of estimated breeding values and SNPs effects. Several genomic regions associated with the level of performance and specific responses of the animals to variations in the gestational environment were revealed, which harbor QTLs and can be exploited for selection purposes. Therefore, genetic evaluation models considering G × Epn and special management and nutrition care for pregnant cows are recommended.

## 1. Introduction

The prenatal environment is recognized as crucial for the development of the bovine fetus and as one of the determining factors of postnatal performance in these animals. Several studies have demonstrated the short- and long-term consequences of developmental programming on growth, reproduction, and offspring health. Some of the most relevant and studied environmental effects during the gestational period include maternal nutrition and thermal environment [1,2,3].

Maternal nutritional restriction during gestation has been associated with various effects, including reduced growth during the prenatal and postnatal periods [1,4,5], decreased muscle fiber formation [6], preferential fetal tissue growth [7], altered placental blood flow and vascularity [8,9], and disrupted reproductive development in both males and females [10,11]. Similarly, thermal stress experienced during gestation impaired the calf’s ability to acquire passive immunity [3,12], altered progeny metabolism [13], reduced birth weight, weaning weight, and animal height [3,12], changed mammary gland morphology [14], and decreased the survival of daughters and milk production for up to three generations [15].

In tropical regions, beef cattle are predominantly raised in grazing systems and exposed to various stressful climatic variables and parasites. In these regions, pregnant cows commonly experience nutritional restriction during the second half of the gestation period, which coincides with the season with low quantity and quality of forage [16]. Therefore, the prenatal environment has a significant economic impact on beef production systems [15,17], especially in tropical regions.

Environmental effects in typical tropical beef cattle production systems commonly interact with animal genotypes [18]. However, little is known about the effects of genotype by prenatal environment interaction (G × Epn) on the postnatal performance of beef cattle, particularly those raised in tropical conditions. According to Holland and Odde [19], two specific interaction environments occur during gestation: the first is the maternal environment, which manifests in uterine interactions with the conceptus; the second is the external environmental effect, mediated by maternal system adaptations to external environmental changes. The way in which the environment influences the future performance of offspring likely involves a complex interaction between the maternal environment, placental alterations, and embryonic epigenetic programming [20].

The present study is designed to identify and characterize the G × Epn on growth and reproduction-related traits in beef cattle raised under tropical conditions. Additionally, we aim to identify genomic regions associated with the level and response in the postnatal performance of the animals to changes in the prenatal environment.

## 2. Materials and Methods

### 2.1. Data Overview

The present study considered two different beef cattle databases of the Nelore breed from Brazil. Both Nelore populations underwent routine genetic evaluations within their respective breeding programs with minimal connection. Therefore, independent analyses were conducted.

The first dataset comprised information from animals born between 1978 and 2018 in an experimental herd (EXP) maintained by the Instituto de Zootecnia, APTA, in Sertãozinho, São Paulo state. Of these animals, approximately 17% were from the control line (animals selected for the post-weaning weight average), 34% were from the line selected for higher post-weaning weight, 44% were from the line selected for higher post-weaning weight and lower residual feed intake, and 5% of the animals were from the founder herd.

The second dataset consisted of a large herd from Agropecuária CFM company (COM) distributed across 12 farms located in the states of Mato Grosso do Sul (49%), São Paulo (45.7%), Bahia (3.7%), and Mato Grosso (1.6%). It is very common for genetic material to be shared among all COM farms, especially in the case of sires, ensuring strong connectivity between farms. The COM animals were born between 1984 and 2019. They were selected over the years for various growth and reproduction-related traits, in addition to utilizing a selection index mainly based on weaning weight, post-weaning weight gain, scrotal circumference, and muscling score.

The animals were kept In high-quality pastures (*Brachiaria* spp., *Panicum* spp.) and received mineral supplements. The breeding season occurred between October and February and lasted 90 days. Heifers were either artificially inseminated or multi-sire natural serviced. The cow-to-bull ratio ranged from 15:1 to 35:1. Calves were kept with their mothers until approximately seven months of age.

### 2.2. Traits

The animals were weighed at birth (BW), around 120 days (W120, only for EXP), and 210 days of age (W210, weaning age). Scrotal circumference (SC), only available for COM, was measured at approximately 550 days of age. For EXP, the days to first calving (DFC) was obtained for all heifers that entered the breeding season, calculated as the difference between the start date of the breeding season and the date of the first calving. Records of heifers that did not calve were included in the analyses by assigning a projected value. This projected value was determined following Johnston and Bunter [21], where the highest DFC record within each management group was identified, and a penalty of 21 days was added to that record. Since the entry date into the breeding season was unavailable for COM animals, the calving date of the first heifer to calve within each management group was identified and assumed as the reference for calculating DFC for all other animals within that group. Thus, the DFC for COM was calculated as the difference between the first heifer’s calving date and the reference date of its respective group, plus 295 days (average gestation length of Zebu animals). Similar to EXP animals, COM heifers that did not calve were included in the analyses by assigning a projected value with a penalty of 21 days.

### 2.3. Genotypes

A total of 1561 EXP animals were genotyped, of which 773 were genotyped using the Illumina BovineHD BeadChip (770 k, Illumina Inc., San Diego, CA, USA), and 788 were genotyped using the GeneSeek Genomic Profiler HDi 75 k (GeneSeek Inc., Lincoln, NE, USA). The genotypes of animals genotyped with the lower-density panel (75 k) were imputed to the high-density (HD) panel using FImpute v.3 software [22]. A dataset containing 6862 animals with HD genotypes was used as the reference population for imputation.

### 2.4. Definition of the Prenatal Environment

**Figure 1:**
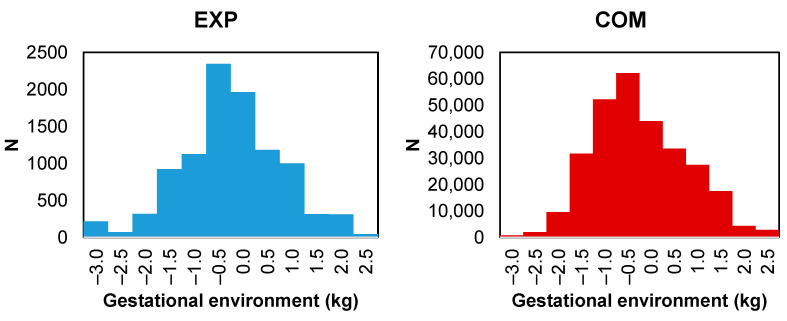
Distribution of the phenotypes of all traits studied according to the gestational environment (in standard deviations) of Nelore cattle from an experimental (EXP) herd and a company (COM).

The objective of this study was not to analyze BW directly; however, this trait was essential for defining the prenatal environment to which the animals were exposed during the entire gestation period. The contemporary group (CG) solutions for BW from each dataset were used as environmental covariates in a reaction norm model in the subsequent stage of the research. The gestational environment was standardized in this study, with a mean of zero and a standard deviation of 1. This standardization allowed for a better comparison and interpretation of the results by eliminating the scale differences between the environmental descriptors of EXP and COM. For EXP, only the CG solutions corresponding to the control line were used in subsequent analyses of all data, as they would more reliably describe the environmental conditions [23]. The CG solutions were obtained through a univariate analysis of BW using a standard animal model. A total of 9816 and 287,705 BW records were analyzed for EXP and COM animals, respectively (Table 1, Figure 1). The CG for BW was defined for EXP by selection line, year, and birth month. For COM, the CG was defined by farm, management group, year, and month of birth. The BW analysis model for each dataset considered the following effects: direct additive genetic effect, maternal additive genetic effect, maternal permanent environmental effect, CG, sex, dam age at calving (covariate), and residual effect. To illustrate the performance of the animals on the phenotypic scale for W120, W210, SC, and DFC according to the previously obtained CG solutions for BW, least squares means were calculated. For this calculation, a model without additive genetic or maternal permanent environmental effects was used, including the corresponding effect of CG for each trait, sex (except for SC and DFC), linear and quadratic dam age at calving (except for DFC), age of the animal at the measurements of W120, W210, and SC, and age at entry into the breeding season for DFC (linear covariate).

### 2.5. Data Quality Control

For the two datasets considered in this study, phenotypes of animals in CG with fewer than five animals, records of animals with an unknown sire or dam, and data exceeding 3.5 standard deviations (except for DFC) above or below the overall trait mean were excluded. For EXP, the CG values for W120 and W210 were defined by selection line, year, and birth month. For SC and DFC, the CG was defined by the selection line and birth year for EXP, and by farm, management group, and birth year for COM. The farm and management group for COM corresponded to those from birth until the time of measurement.

Quality control of genomic data was performed, retaining only autosomal single nucleotide polymorphisms (SNPs) with minor allele frequencies > 0.02, the *p*-value for the Hardy–Weinberg equilibrium > 10^−5^, a call rate > 92% for SNPs, and a call rate > 90% for samples. After quality control, the total number of SNPs was 383,570. The overall description of the data after quality control is presented in Table 1.

### 2.6. Multiple-Trait Reaction Norm Model

A reaction norm model was applied separately in multiple-trait analyses to each dataset. The adopted animal model can be described as follows:

$$y=X\beta +Z_{di}d_{i}+Z_{ds}d_{s}+Z_{mi}m_{i}+Z_{ms}m_{s}+Ppm+e$$

where y is the vector of observations; β is the vector of systematic effects of CG, sex (W120 and W210), age at measurement as linear covariate (W120, W210, and SC), age at the beginning of the breeding season as linear covariate (DFC), dam’s age at calving as a linear and quadratic covariate (except for DFC); $d_{i}$ is the vector of direct additive genetic intercepts of reaction norms; $d_{s}$ is the vector of direct additive genetic slopes of reaction norms; $m_{i}$ is the vector of maternal additive genetic intercepts of reaction norms (except for SC and DFC); $m_{s}$ is the vector of maternal additive genetic slopes of reaction norms (except for SC and DFC); pm is the vector of maternal permanent environment effects (except for SC and DFC); and e is the residual vector. For simplicity of multiple-trait analyses, the residual variance was assumed to be homogeneous along the environmental gradient. X, $Z_{di}$, $Z_{ds}$, $Z_{mi}$, $Z_{ms}$, and P were incidence matrices, where $Z_{ds}$ and $Z_{ms}$ included the prenatal environment descriptor covariate (as previously defined) and related y to the corresponding vectors $d_{s}$ and $m_{s}$. For W120 and W210 (a more complex model), it was assumed that:

$$\left[\begin{array}{c} d_{i}\\ d_{s}\\ m_{i}\\ m_{s} \end{array}\right]\sim Ν(0,\left[\begin{array}{cccc} \sigma_{d_{i}}^{2} & \sigma_{d_{is}} & \sigma_{d_{i}m_{i}} & \sigma_{d_{i}m_{s}}\\ \sigma_{d_{is}} & \sigma_{d_{s}}^{2} & \sigma_{d_{s}m_{i}} & \sigma_{d_{s}m_{s}}\\ \sigma_{d_{i}m_{i}} & \sigma_{d_{s}m_{i}} & \sigma_{m_{i}}^{2} & \sigma_{m_{is}}\\ \sigma_{d_{i}m_{s}} & \sigma_{d_{s}m_{s}} & \sigma_{m_{is}} & \sigma_{m_{s}}^{2} \end{array}\right]⨂A)$$

where $\sigma_{d_{i}}^{2}$ and $\sigma_{m_{i}}^{2}$ are intercept variances for direct and maternal additive genetic effects, respectively; $\sigma_{d_{s}}^{2}$ and $\sigma_{m_{s}}^{2}$ are slope variances for direct and maternal additive genetic effects, respectively; ⨂ is the product of Kronecker; and A is the numerator relationship matrix between animals considering pedigree information. For EXP, A matrix was replaced by the H matrix that combines pedigree and genomic information [24]. This approach is known as single-step genomic reaction norm model. The residual vector corresponding to each trait was assumed to be $Ν\sim (0,\;I\sigma_{e}^{2})$, where I is an identity matrix. Using the Gibbs sampler, conditional Gaussian distributions of systematic effects, breeding values, and inverted Wishart distributions for genetic and residual (co)variances were sampled.

Samples of the posterior distributions of the covariance components were obtained using the GIBBS2F90 program [25]. Chains of 550,000 samples were obtained, with a burn-in of 100,000 samples and sampling of covariance component estimates every 50 cycles. Convergence was evaluated through visual inspection and the Geweke test [26].

### 2.7. Single-Step Genomic-Wide Association Study (ssGWAS)

The effects of markers on the intercept and slope of the reaction norms for each trait were estimated using the weighted single-step GBLUP method (scenario S1 with two iterations) proposed by Wang et al. [27]. The percentage of genetic variance explained by moving genomic windows of five adjacent SNPs was obtained using the postGSf90 program [28]. Genomic windows that explained at least 0.5% of the genetic variance for the Intercept or slope of each trait were considered potentially associated with the overall level of performance and the specific response of animals to changes in the prenatal environment. Genes within the candidate genomic regions were annotated using the Ensembl Genes 103 database (www.ensembl.org/index.html, accessed on 22 March 2021) and the ARS-UCD1.2 bovine genome assembly [29]. Additionally, the QTLdb database for cattle (https://www.animalgenome.org/cgi-bin/QTLdb/BT/index, accessed on 22 March 2021; [30]) was explored to determine if any candidate genomic regions had been previously reported as quantitative trait loci (QTL) in cattle.

### 2.8. Reaction Norms

The top 0.5% SNPs in EXP were sampled for each effect and trait in the most unfavorable and favorable environments to illustrate the G × Epn on the studied traits at the SNP level. Thus, the reaction norms of each SNP group were presented along the environmental gradient in terms of the percentage of genetic variance explained. Additionally, 100 EXP and COM bulls (with at least 25 progeny records) were randomly sampled, and their reaction norms were displayed along the scale of gestational environment values.

## 3. Results

### 3.1. Effect of Gestational Environment on the Phenotypic Scale

**Figure 2:**
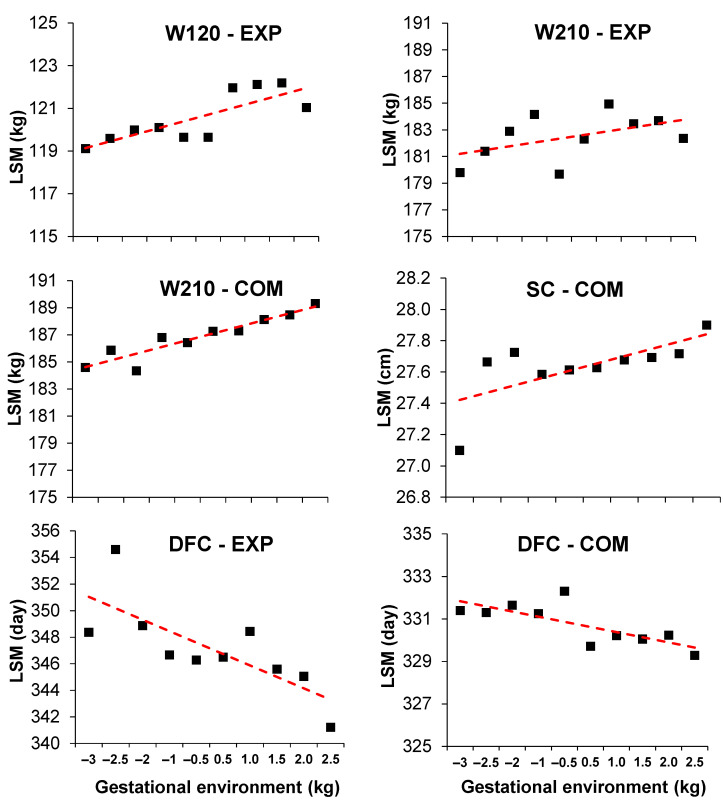
Least squares means (LSM) of body weight at around 120 (W120) and 210 (W210) days of age, scrotal circumference (SC), and days to first calving (DFC) according to the gestational environment (in standard deviations) of Nelore cattle from an experimental (EXP) herd and from a company (COM).

The animals showed a variation in performance across the gestational environment (CG solutions for BW) for all studied traits (Figure 2). The W120 and W210 from the EXP herd exhibited an average increase of 0.518 and 0.469 kg for each 1 standard deviation unit of gestational environment, respectively. In the COM herd, a 0.820 kg increase per 1 standard deviation unit of gestational environment was observed for W210. These changes represented a variation of up to 2.61% and 1.56% in the average performance of EXP animals for W120 and W210, respectively, along the environmental gradient. For COM, a variation of up to 2.66% in the average performance of animals for W210 was observed along the considered environmental gradient. A slight increase was observed in SC for COM as the gestational environment became more favorable (0.077 cm/standard deviation of gestational environment). For this trait, an average variation of up to 1.68% in animal performance was observed throughout the considered environments. The DFC of EXP animals was reduced by 1.42 days for each unit increase in the standard deviation of the gestational environment. In this regard, an average variation of up to 2.5% in the DFC values of animals was observed along the environmental gradient. For COM, DFC was modestly reduced for higher values of the gestational environment (−0.40 days per standard deviation unit).

### 3.2. Covariance Components and Genetic Parameters

The mean genetic correlations between the intercept and slope of reaction norms for direct and maternal effects ranged from −0.073 (DFC) to 0.215 (W120) for the studied traits in the EXP herd (Table 2). These estimates were generally higher for the COM herd, ranging from 0.313 (SC) to 0.607 (W210). On average, the slope variance for direct and maternal effects represented between 0.029 (SC) and 0.651 (DFC) of the variance associated with the intercept in the studied herds.

### 3.3. Heritability Estimates

**Figure 3:**
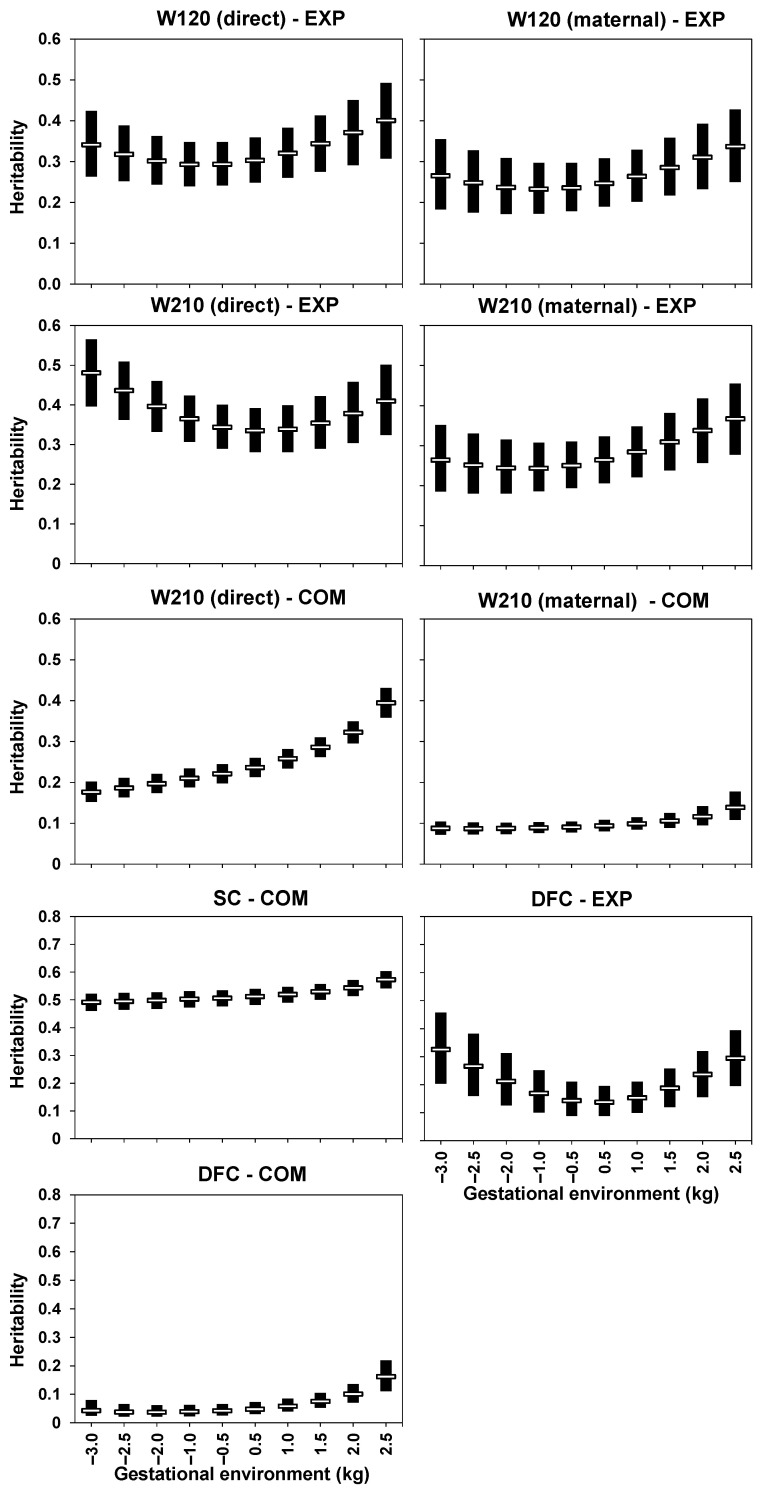
Posterior mean of the heritability estimates (hollow horizontal bar) and high posterior density interval 95% (solid vertical bar) of body weight at around 120 (W120) and 210 (W210) days of age, scrotal circumference (SC), and days to first calving (DFC) of Nelore cattle from an experimental (EXP) herd and from a company (COM) according to the gestational environment (in standard deviations).

Heritability estimates exhibited variations to a greater or lesser extent along the gestational environment for all studied traits and herds (Figure 3). The mean estimates of heritability for W120 ranged from 0.29 to 0.40 for direct effects and 0.23 to 0.34 for maternal effects in the EXP herd. For W210 in the EXP herd, the mean estimates ranged from 0.33 to 0.48 for direct effects and from 0.24 to 0.37 for maternal effects. In the COM herd, the posterior means of the heritability estimates for W210 ranged from 0.18 to 0.39 for direct effects and from 0.09 to 0.14 for maternal effects along the gestational environment gradient. The mean estimates of heritability for SC in the COM herd were high across all levels of the environmental gradient (0.49 to 0.57). In contrast, the mean estimates of heritability for DFC varied considerably across different levels of the gestational environment, ranging from 0.14 to 0.32 in EXP and from 0.04 to 0.16 in COM.

### 3.4. Intra-Trait Genetic Correlations

The genetic correlations for the direct and maternal effects of W120 and W210 showed considerably different estimates from unity between the distant values of the gestational environment (Table 3). These estimates reached values below 0.40 for W120 and W210 in the EXP herd. For COM, the estimates were up to 0.722 and 0.640 for direct and maternal effects of W210, respectively. DFC exhibited the lowest mean posterior estimates among all studied traits, with values of up to −0.294 in EXP and 0.102 in COM. On the other hand, SC in the COM herd consistently showed high mean estimates of genetic correlation (>0.89) across all considered environments.

### 3.5. Inter-Trait Genetic Correlations

**Figure 4:**
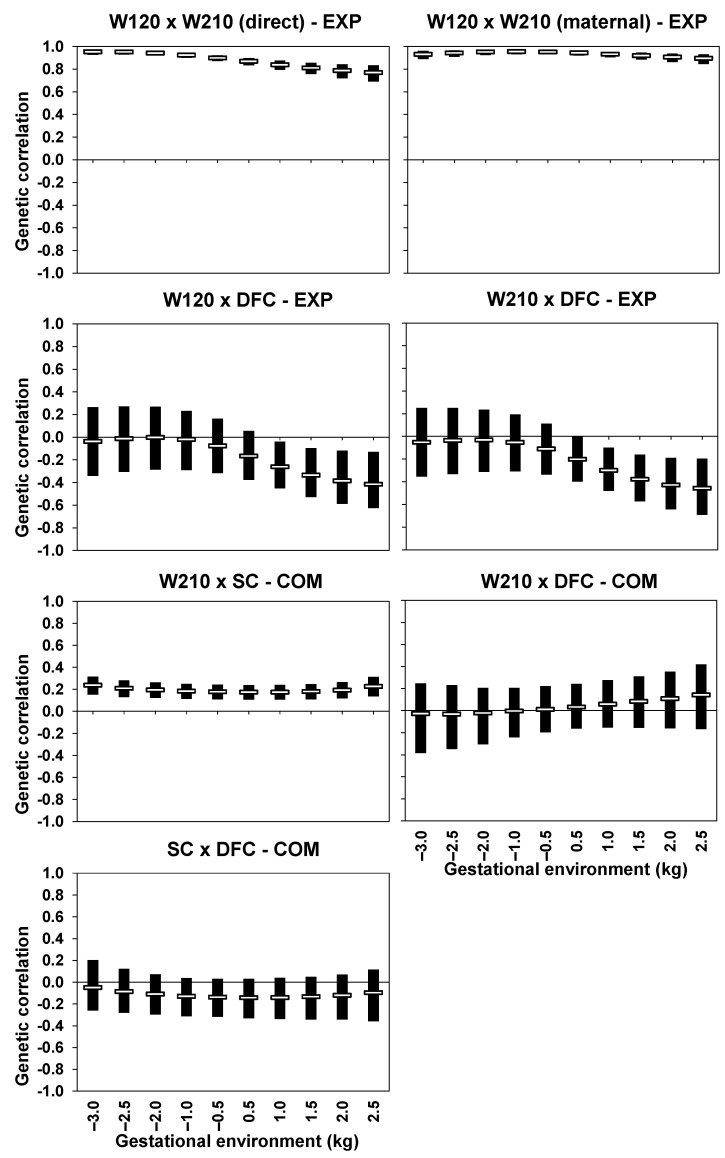
Posterior mean of the genetic correlation estimates (hollow horizontal bar) and high posterior density interval 95% (solid vertical bar) between body weight at around 120 (W120) and 210 (W210) days of age, scrotal circumference (SC), and days to first calving (DFC) of Nelore cattle from an experimental (EXP) herd and from a company (COM) according to the gestational environment (in standard deviations).

The mean posterior estimates of genetic correlations between W120 and W210 in EXP were consistently high, exceeding 0.77 and 0.89 for direct and maternal effects, respectively (Figure 4). The genetic correlations between W120-DFC and W210-DFC in EXP followed the same pattern along the environmental gradient. These estimates were close to zero (−0.04) in less favorable gestational environments and tended to become stronger and negative (around −0.43) in more favorable environments. In COM, the mean posterior estimates of genetic correlation between W210 and SC were around 0.20 across the entire range of the environmental gradient, with a slight tendency to decrease in intermediate environments. The genetic correlations between W210 and DFC in COM were slightly negative (−0.03) or close to zero in lower and intermediate environments but tended to be slightly positive in higher environments (0.14). The mean estimates of genetic correlation between SC and DFC for the COM herd were consistently negative, ranging from −0.05 to −0.14, indicating a favorable relationship across all values of the environmental gradient.

### 3.6. ssGWAS

**Figure 5:**
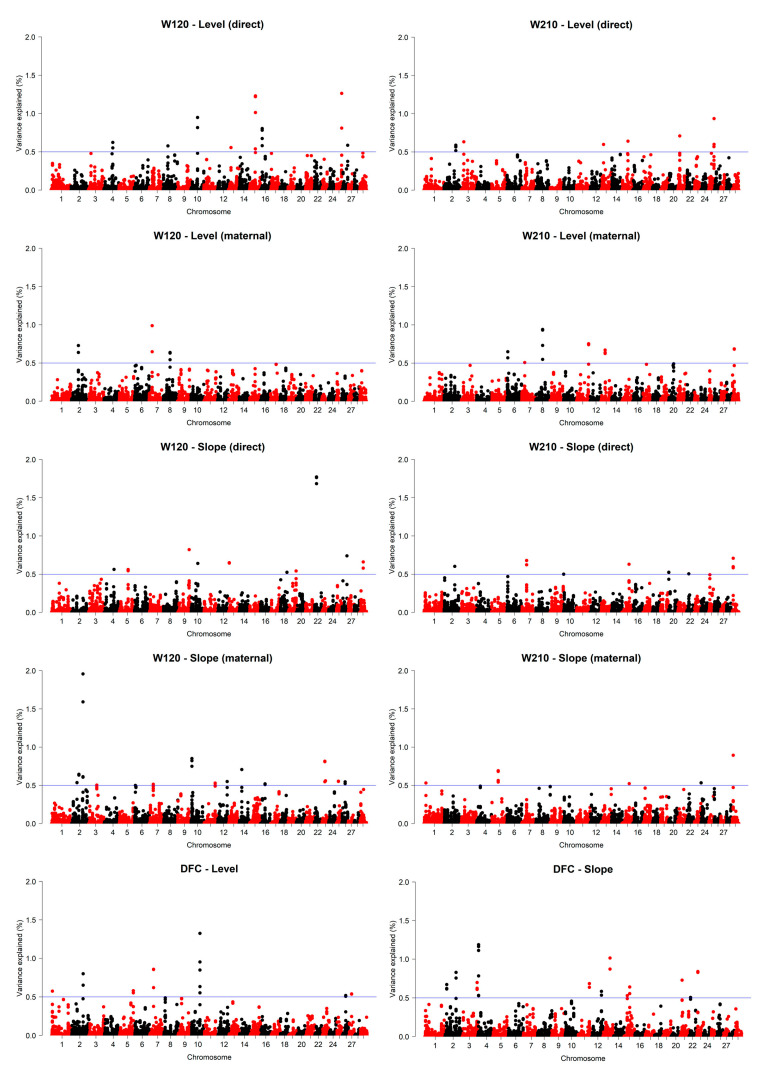
Manhattan plots for the percentage of variance explained for the level and slope of the reaction norms of body weight at around 120 (W120) and 210 (W210) days of age, scrotal circumference (SC), and days to first calving (DFC) of Nelore cattle from an experimental herd.

The genomic windows that explained at least 0.5% of the total genetic variance for the level and slope of the reaction norms for direct and maternal effects were spread across all autosomal chromosomes except BTA 17, 27, and 28 (Figure 5). The top three genomic windows that explained the highest percentage of the total genetic variance for the level and slope of the reaction norms for each trait are presented in detail in Table 4 and Table 5. The highest peaks for the intercept of the direct effects of W120 and W210 were located on BTA 25 (40.6 to 41.0 Mb) and explained 1.26% and 0.94% of the genetic variance for this component, respectively. This region contained genes related to residual feed intake, conception rate, milking speed, and average daily weight gain. Another common genomic window between W120 and W210 for the intercept (direct effect) was found on BTA 15, where genes previously associated with milk-fat production and weight gain were located. For the maternal effect, the highest peaks of explained variance were located on BTA 7 (21.9 to 22.3 Mb) and BTA 8 (52.7 to 53.1 Mb) for W120 and W210, respectively. Both regions harbored genes involved in milk production and quality, disease susceptibility, reproductive performance, and productive life. The genomic window that explained the highest percentage of the total genetic variance for the intercept of the reaction norms for DFC was found on BTA 10 (66.5 to 66.9 Mb) and explained 1.32% of the variance for this component. This region contained genes primarily associated with milk production, quality, udder traits, stature, and disease susceptibility. Genomic windows on BTA 7 and 2 were also found for the intercept of DFC. They contained genes associated with health, birth weight, body capacity, milk traits, and pregnancy rate of the animals.

For the slope of the reaction norms of the direct effect (Table 5), the regions located on BTA 22 (17.3 to 17.7 Mb) and BTA 29 (67.3 to 71.3 Mb) were the ones that showed the highest peak of explained genetic variance. These regions harbored genes, such as SRGAP3, RAD18, and GRM5, related to body weight, milk composition and quality, lean-meat yield, and shear force. Relevant genomic regions were identified on BTA 9 and 15, which contained genes, such as 7SK, MYCT1, VIP, RPS3, SNORD15, KLHL35, GDPD5, SERPINH1, MAP6, and MOGAT2, involved in udder and teat functional traits, muscle composition, milk production, reproductive performance, and disease susceptibility. For the maternal reaction norms slope, the highest peak of explained genetic variance was found on BTA 2 (89.2 to 89.6 Mb) and harbored genes associated with conception rate, intramuscular fat, and milk-fat yield for W120. The same region on BTA 29 (67.3 to 71.3 Mb) identified for the direct effect of W210 was also found to be the most important in terms of explained variance for the maternal effect of this trait. Genomic windows on BTA 11 (76.4 to 76.8 Mb), 2 (111.96 to 112.23 Mb), and 1 (149.05 to 149.4 Mb) were identified as important for the slope of the DFC reaction norms. Among the genes in these regions, those related to reproductive performance, productive life, body weight, and heat tolerance can be highlighted.

Among all the important genomic regions found, nine were exclusively related to the slope of the reaction norms and explained 6.83% of the genetic variance for the direct effect of W120. For W210, six genomic windows associated exclusively with the reaction norms’ slopes were identified and explained 3.65% of the genetic variance for the direct effect. For the maternal effect, 14 and five genomic windows exclusively related to the slope of the reaction norms explained 9.78% and 3.17% of the genetic variances for W120 and W210, respectively. For DFC, nine genomic windows exclusively related to the reaction norms’ slopes were identified and explained 6.99% of the genetic variance for this component.

### 3.7. Reaction Norms

**Figure 6:**
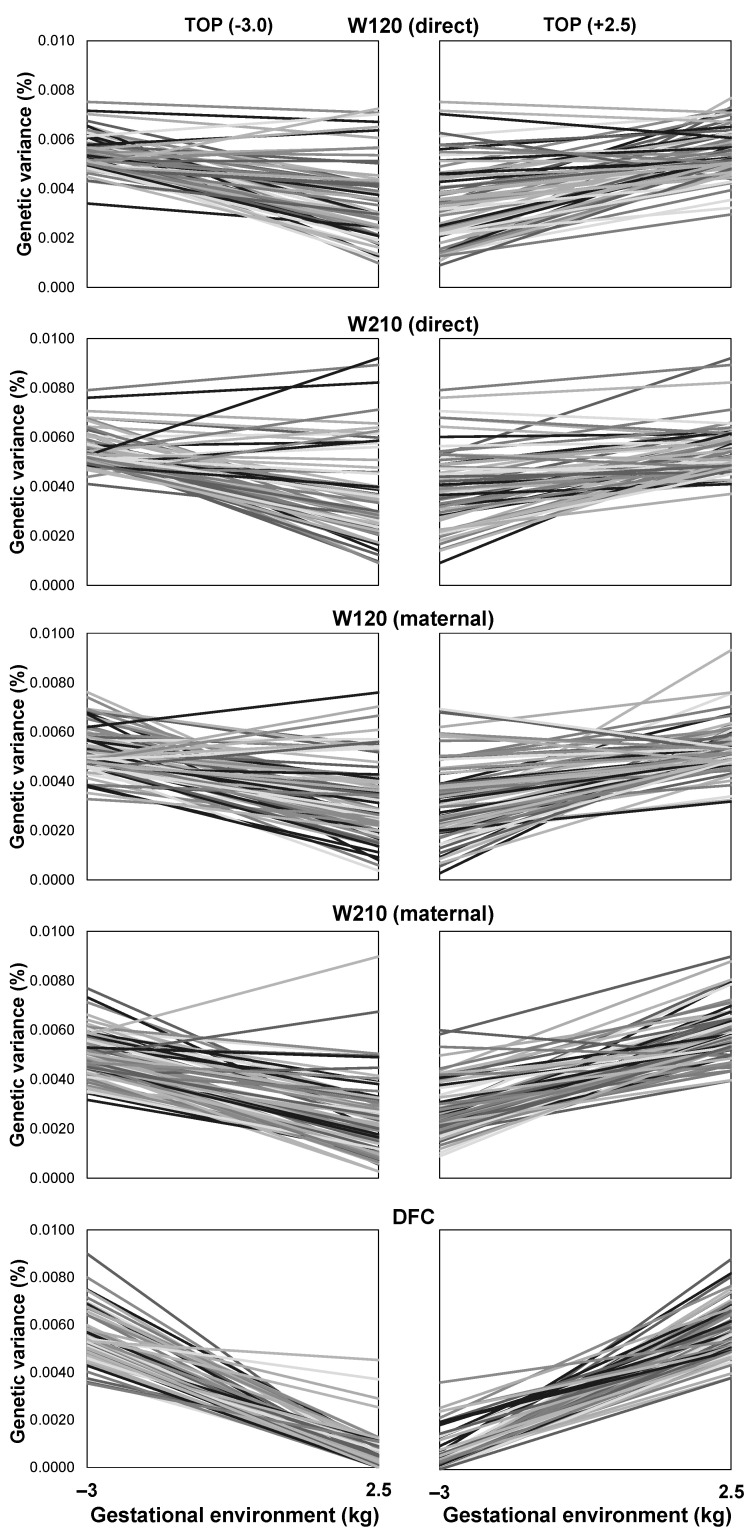
Top 0.5% SNPs sampled based on the genetic variance explained according to the gestational environment for body weight at around 120 (W120) and 210 (W210) days of age and days to first calving (DFC) in an experimental herd of Nelore cattle under the most unfavorable and favorable gestational environments.

A substantial SNP × prenatal environment interaction was observed for all the studied traits in the EXP herd (Figure 6). Some SNPs explained a considerable percentage of the genetic variance for each trait across distant environments, indicating a persistent effect across different environments. However, most SNPs had their importance in terms of explained variance dependent on the quality of the prenatal environment. In other words, most SNPs that explained a higher percentage of the genetic variance in one environment explained little in another, especially for DFC.

**Figure 7:**
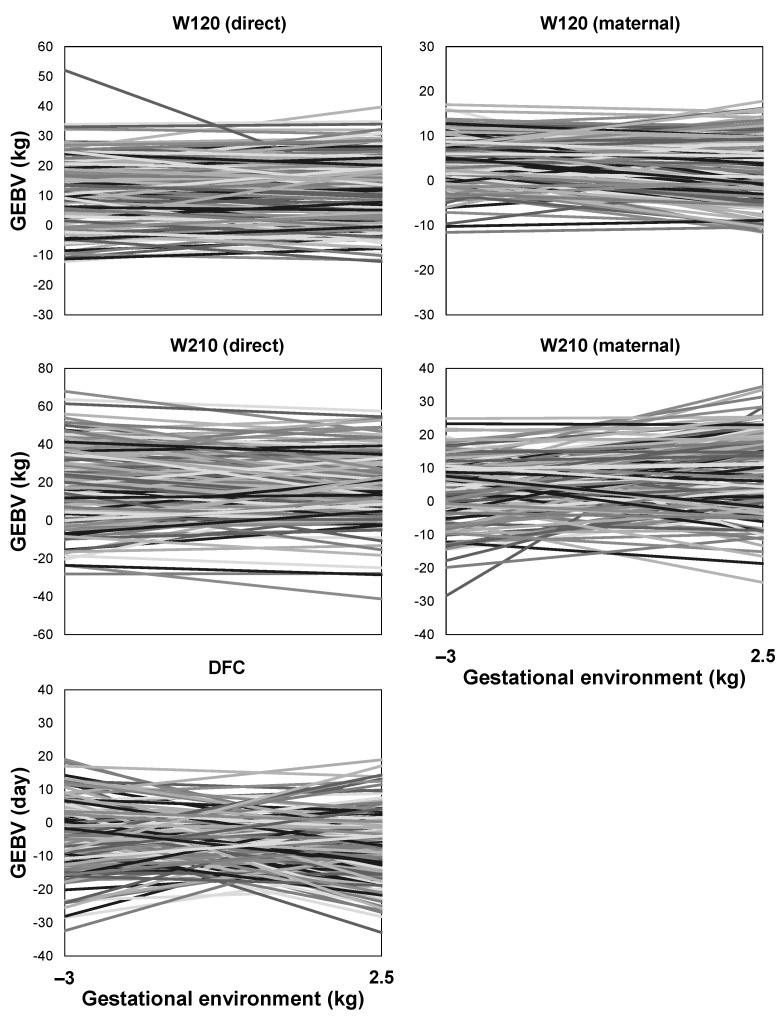
Estimated direct genomic breeding values (GEBVs) on the gestational environment for a sample of 100 Nelore sires from an experimental (EXP) herd for body weight at around 120 (W120) and 210 (W210) days of age and days to first calving (DFC).

**Figure 8:**
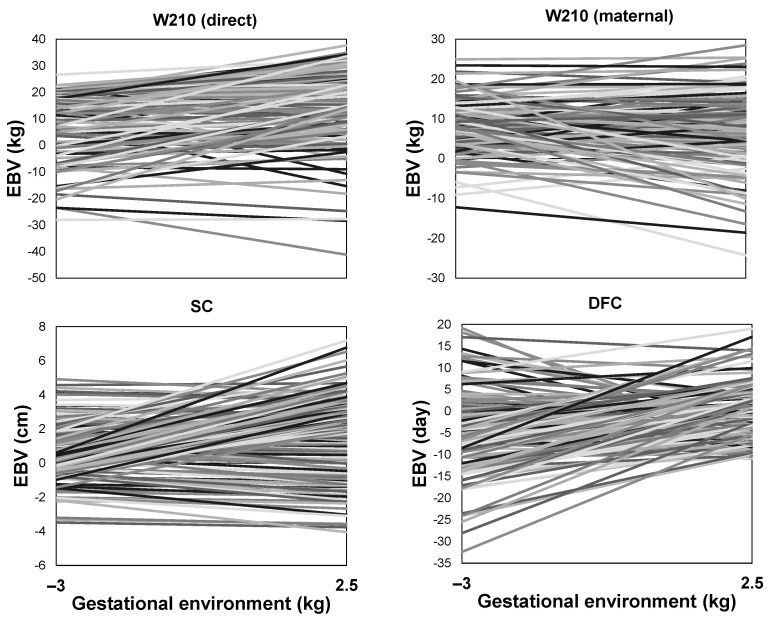
Estimated direct breeding values (EBVs) on the gestational environment for a sample of 100 Nelore sires from a company (COM) herd for body weight at around 210 days (W210) of age, scrotal circumference (SC), and days to first calving (DFC).

The reaction norms of bulls from the EXP and COM herds along the environmental gradient demonstrated a substantial re-ranking of estimated breeding values (Figure 7 and Figure 8). Both highly plastic animals (showing high sensitivity of their estimated breeding values to the environment) and robust animals (exhibiting stable estimated breeding values across different values of the environmental gradient) were identified. 

## 4. Discussion

The primary motivation of the present study was that beef cattle raised in tropical pasture environments, such as those in Brazil, are subjected to breeding seasons ranging from October to February (rainy season in the southern hemisphere). Therefore, calves are typically born between July and October (dry season in the southern hemisphere), which is an appropriate time for early calf care (umbilical cord healing, colostrum intake, and nursing). On the other hand, pregnant cows experience the final stage of gestation during the dry season, when the quality and quantity of forage are typically limited. If appropriate supplementation is not provided during this period, cows inevitably experience nutritional restriction, which can affect the development of their calves in utero. Additionally, some heat stress can be experienced by cows and their offspring, as regions, such as central Brazil (where some of the animals in this study were raised) commonly have temperatures above 30 °C even during winter (June to September).

The literature evidence indicates that intrauterine growth retardation can lead to slower growth throughout the life of cattle [1,4]. In this regard, our results consistently show that poorer gestational environments result in more modest growth, smaller scrotal circumference, and delayed days to calving in the progeny of beef cattle. Indeed, the impact of gestation environment was not only evident in the phenotypic performance of the offspring but also at the genetic level. Roberts et al. [31], in an extensive study on beef heifer development and lifetime productivity in rangeland-based production systems, observed that cows subjected to dietary restrictions and born to marginally supplemented mothers produced lighter calves at birth and weaning compared to their contemporaneous herd mates born to adequately supplemented mothers. Similarly, Greenwood et al. [4] demonstrated that fetal growth restriction (reduced birth weight) can limit the ability of cattle to exhibit compensatory growth. Thus, the offspring of mothers experiencing nutritional restriction during the final stage of gestation showed lower weight and weight gain outcomes at any postnatal age compared to their better-nourished counterparts. In line with these studies, Robinson et al. [32] indicated that fetal growth retardation and reduced birth weight led to reductions in weight at feedlot exit, hot carcass weight, and retail yield. Additionally, maternal nutrition can affect the ovarian reserve, testicular development, prepubertal reproductive development, and attainment of puberty in beef cattle [33,34,35,36]. In this sense, fetal growth impairments can have important economic implications for beef cattle production systems, particularly those based on pasture.

Taking the ratio between the slope and intercept of reaction norms as a measure of the magnitude of G × Epn, SC exhibited low, body weight traits exhibited moderate, and DFC exhibited high G × Epn values. These findings are similar to those reported by Chiaia et al. [37] for Nelore cattle. Chiaia et al. [37] reported a strong genotype by environment interaction effect for age at first calving compared to the results observed for SC or yearling weight. Santana et al. [38] indicated that SC in Nelore animals is a less plastic trait. Therefore, a reduced re-ranking of breeding values can be expected for SC along the environmental gradient compared to traits, such as post-weaning weight gain.

Consistent with the magnitude of G × Epn discussed above, while SC exhibited relatively stable heritability estimates along the environmental gradient, the other traits studied showed substantial variation in their estimates. In this sense, selection responses can be considerably different depending on the prenatal environment to which the animals are exposed. Hay and Roberts [39] also observed differences in variance and heritability estimates between adequate and marginal prenatal nutritional environments for beef cattle. These authors found higher heritability estimates for birth, weaning, and yearling weights in a marginal prenatal environment compared to a nutritionally adequate environment for pregnant cows. Hay and Roberts [39] argued that this result could be because more favorable environments can mask the animals’ true genetic potential or differences in the adaptation of the animals used in the experiment to the prenatal environments studied. Despite the differences between the study by Hay and Roberts [39] and ours, the results found here indicate that, in general, better prenatal environmental conditions lead to higher heritability estimates in the studied population.

A general trend of higher heritability estimates was observed for extreme gestational environments, while lower estimates were found near the zero environment. This behavior can be explained by at least two reasons. The first factor is the smaller number of observations in extreme environments, which leads to less accurate estimates of genetic parameters at the ends of the evaluated environmental scale. This is evident through the larger standard deviations obtained in the extreme environments. The second factor seems related to the point on the environmental gradient where a higher number of interceptions of linear reaction norms occur. In other words, some animals show an improvement, while others show a decline in terms of genetic merit, as observed by Alves et al. [40].

Except for SC, all genetic correlation estimates for within-trait direct and maternal effects were below unity in distant discrepant environments. DFC exhibited the lowest estimates of genetic correlation between distant environments, as it was the trait that showed the highest environmental sensitivity and the greatest G × Epn effect. In general, the standard deviations associated with the estimates of genetic correlation within traits were relatively small compared to the mean, indicating that the true value of the parameter in question was likely to fall within a narrow interval with high probability. DFC showed higher standard deviations due to the smaller number of records available for this trait. When the genetic correlation between two character states deviated from unity, it indicated that phenotypes in each environment were influenced by different alleles or by the same alleles in different ways, suggesting the possibility of independent evolution [41]. Therefore, selection practices for a given trait in one environment may not yield the desired results in another. Hay and Roberts [39] consistently found strong direct genetic correlations (≥0.97) between two prenatal nutritional environments. However, they observed maternal genetic correlations ranging from 0.41 to 0.73 for birth, weaning, and yearling weights in a composite beef cattle breed, indicating maternal genetic and prenatal nutritional interaction effects. Hay and Roberts [42] also reported consistent genetic correlations below unity for post-weaning average daily gain, yearling weight, intramuscular fat percentage, and 12th rib fat depth across different gestational and post-weaning environments. Therefore, considering pre- and postnatal production environments is important for genetic evaluations of beef cattle, especially those raised in pasture-based systems.

Inter-trait genetic correlations showed varying degrees along the environmental gradient for all traits, with the strongest correlations observed between W120 and DFC and between W210 and DFC in EXP. The posterior means of the genetic correlation estimates between W120 and W210 were similar to those reported by Boligon et al. [43] for direct (0.81) and maternal (0.79) effects between W120 and W240 in a Nelore cattle population. These estimates suggest that W120 and W210 are influenced by the same genes in different gestational environments. The genetic correlations between W120 and DFC and between W210 and DFC in EXP were favorable in more favorable gestational environments. In contrast, a modest trend toward a less favorable genetic association between W210 and DFC was observed in more favorable gestational environments in COM. It is worth emphasizing that the two studied Nelore cattle populations have different structures, quantities of analyzed information, management practices, and geographic location. Therefore, differences in genetic parameters are naturally expected. Thus, different management and selection strategies should be developed to optimize genetic progress in the two studied populations. In a study with Nelore cattle, Chiaia et al. [37] observed that genetic correlations between yearling weight and age at first calving ranged from −0.05 to −0.32 along the environmental gradient adopted by those authors. Chiaia et al. [37] reported that genetic correlation estimates between yearling weight and age at first calving were more favorable when more favorable production environments were provided for yearling weight and less favorable for age at first calving. All these results demonstrate the complexity of the subject matter in the present study and how the genetic mechanisms underlying the association between growth and reproductive traits in cattle can vary depending on the imposed environmental conditions.

The posterior means of the genetic correlation estimates between W210 and SC were relatively low and showed slight variations across different gestational environments. Santana et al. (2015) reported relatively close genetic correlation estimates (0.03 to 0.20) between post-weaning weight gain and SC for Nelore cattle in different production environments. The means of the genetic association estimates between SC and DFC in COM were consistently negative and favorable for all gestational environments. This finding is similar to that Chiaia et al. [37] reported between SC and age at first calving in Nelore cattle along the environmental gradient (−0.14 to −0.60). It is important to note that the environmental gradients adopted by Chiaia et al. [37] and Santana et al. [38] differed from those used in the present study, hindering the direct comparison of results. Nevertheless, the results obtained here suggest that selection practices for SC in any environment, especially in intermediate and favorable environments, can contribute to some extent to the improvement of female reproductive performance.

The behavior of reaction norms at the level of (G)EBV or SNP for all traits demonstrated, to a greater or lesser degree, the presence of G × Epn. The re-ranking of breeding values for Nelore cattle raised in pasture-based systems has been consistently reported in the literature [23,44,45]. Based on the approach adopted in the present study, epigenetic effects through fetal programming can be partially responsible for animals’ phenotypic and genetic responses to the gestational environment for the analyzed traits. Fetal programming induced by maternal nutrition during gestation can affect the expression of genes related to reproductive and growth traits in beef cattle [46,47,48]. Polizel et al. [48] found evidence of G × Epn and explained that the results obtained could be attributed to epigenetic mechanisms resulting in changes in response to environmental adaptations. Other factors, such as thermal stress, have been reported as determinants of fetal programming and can affect the future performance of bovine offspring [14]. In the present study, we believe that nutrition and thermal stress, especially, may be responsible for inducing differential genetic responses in animals. However, we did not exclude other factors related to maternal health, such as parasites and diseases.

The approach adopted here to describe the quality of the gestational environment did not allow for separately determining the factors that were the most relevant to the future performance of progeny. On the other hand, contemporary groups represent the most elementary entities for characterizing the production environments of beef cattle [49]. This approach provided a comprehensive representation of the environmental conditions throughout the gestational period, as the contemporary group solution for BW could capture many of the environmental stimuli and insults that affected fetal development. Unlike other studies that focus only on specific time points during gestation, our analysis considered the entire gestational period. It is worth noting that there is a knowledge gap regarding the effects of maternal nutrition, especially during the early stages of gestation and in Zebu animals, which highlights the need for further research on fetal programming, as pointed out by Barcelos et al. [50].

The GWAS revealed important regions associated with the level of performance and specific responses of animals to variations in the quality of the gestational environment. The published bovine QTL database allowed us to identify several genomic regions that overlapped with previously related regions harboring QTLs that influenced milk quality, growth, meat characteristics, adaptation, health, productive life, and reproduction of animals. Not surprisingly, for W120 and W210, common candidate genes were identified due to the close additive genetic relationship between these traits. The genes GNA12 and AMZ1 on BTA25 were reported as important for carcass gain in Holsteins (Mao et al., 2016). AMZ1, BRAT1, and PRUNE2 affected residual feed intake and maintenance efficiency in Nelore cattle [51,52]. For example, BRAT1 regulates cell growth and apoptosis [53]. The IRF1 gene on BTA 7, identified for the maternal effect intercept of W120, was associated with age at first corpus luteum, post-partum anestrous interval, and post-partum anestrous interval in Brahman and Tropical Composite cows [54]. The genes OR2D2, OR2D3, OR10A4, ZNF214, and ZNF215 located on BTA15 were identified as important for dry matter intake in Nelore cattle from the same experimental herd studied here [55]. The GDPD5 gene was recognized for the slope of the direct effect of W210. A significantly hypermethylated site within the gene body region of GDPD5 in prenatally stressed Brahman calves was previously identified [56]. Thus, the differential methylation of GDPD5 may influence biological processes in prenatally stressed calves.

For DFC, genes, such as CDKN3, AOX2, AOX4, and BZW1, related to bovine health and reproduction were identified for the intercept of reaction norm. The cyclin-dependent kinase inhibitor 3 gene (CDKN3) was associated with metritis in first-lactation Holstein cows. BZW1 was significantly induced in the bovine small intestine by *Cooperia oncophora* infections [57]. In Brazil, most parasites recovered from pasture-raised cattle belong to the genus *Cooperia*. Additionally, Hoelker et al. [58] showed that the downregulation of BZW1 could influence the dynamic progression of embryos from cattle with subclinical endometritis. Furthermore, the genes AOX2 and BZW1 overlapped with health-related QTLs in Shanghai Holstein cattle [59].

For the slope of the reaction norm of DFC, the SERPINE2 gene was identified. This gene was reported as an important protease inhibitor in growing follicles and corpora lutea of crossbred heifers [60]. In this regard, Bédard et al. [60] suggested that the high expression of SERPINE2 may contribute to follicular growth. The PIGP gene has been previously associated with sperm motility in Italian Holstein bulls by Ramirez-Diaz et al. [61] and with fatty acid composition of adipose tissue in Australian beef cattle breeds [62]. Another candidate gene, HLCS, has been previously associated with beef production and carcass quality traits in Korean native cattle breeds [63].

## 5. Conclusions

Substantial genotype by prenatal environment interaction has been identified for traits related to the growth and reproduction of beef cattle raised under tropical grazing conditions. This interaction was strong enough to result in heterogeneity of variance components and genetic parameters in addition to re-ranking of estimated breeding values and SNPs effects. Therefore, pregnant cows’ nutrition, health, and well-being can affect the development of the bovine fetus and the offspring’s future productive and reproductive performances. Genetic evaluation models considering genotype by prenatal environment interaction and special management and nutrition care for pregnant cows are recommended.

Several genomic regions associated with the level of performance and specific responses of the animals to variations in the quality of the gestational environment were revealed. These regions overlapped with previously identified regions harboring QTLs that influence economically important traits in cattle and can be further explored for selection purposes.

## Figures and Tables

**Table 1:** Summary of the data structure for birth weight (BW), body weight at around 120 (W120) and 210 (W210) days of age, scrotal circumference (SC), and days to first calving (DFC) of Nelore cattle from an experimental herd and a company.

Item	Experimental Data				
	BW (kg)	W120 (kg)	W210 (kg)	SC (cm)	DFC (days)
Animals in the pedigree, n	10,350	9614	9573	–	2969
Sires in the pedigree, n	384	384	384	–	341
Dams in the pedigree, n	2540	2470	2469	–	1452
Phenotypic records, n	9816	9078	9003	–	2222
Sires with progeny record, n	370	370	370	–	306
Dams with progeny record, n	2519	2446	2447	–	1289
Genotyped sires with progeny record, n	116	116	116	–	70
Genotyped dams with progeny record, n	370	365	366	–	158
Progeny records from genotyped sire, n	3328	3077	3058	–	572
Progeny records from genotyped dams, n	1299	1199	1194	–	199
Genotyped animals with phenotypic records, n	1516	1503	1502	–	258
Average of the trait	28.92	122.91	188.99	–	347.80
Standard deviation of the trait	5.43	19.94	31.02	–	35.81
	Company data				
Animals in the pedigree, n	356,730	–	216,707	120,619	25,072
Sires in the pedigree, n	3060	–	2476	2022	1318
Dams in the pedigree, n	133,863	–	99,553	67,291	35,950
Phenotypic records, n	287,705	–	146,020	52,259	22,405
Sires with progeny record, n	2342	–	1817	1418	765
Dams with progeny record, n	95,820	–	65,379	33,588	17,391
Average of the trait	31.87	–	188.65	27.62	340.47
Standard deviation of the trait	3.96	–	27.53	3.28	27.22

**Table 2:** Posterior mean, standard deviation (SD), 95% highest posterior density interval (HPD) of (co)variance components, genetic parameters of the reaction norm model applied to the body weight at around 120 (W120) and 210 (W210) days of age, scrotal circumference (SC), and days to first calving (DFC) of Nelore cattle from an experimental herd and from a company.

Trait	Parameter	Experimental Data			Company Data		
		Mean	SD	HPD95%	Mean	SD	HPD95%
W120	$\sigma_{d}^{2}$	107.633	12.570	83.951 to 133.148	–	–	–
	$\sigma_{m}^{2}$	87.426	13.384	62.855 to 115.186	–	–	–
	$\sigma_{pm}^{2}$	53.373	5.747	42.060 to 64.750	–	–	–
	$c^{2}$	0.166	0.019	0.129 to 0.202	–	–	–
	$\sigma_{e}^{2}$	110.854	4.826	101.550 to 120.200	–	–	–
	$r_{d_{is}}$	0.177	0.094	−0.016 to 0.353	–	–	–
	$r_{m_{is}}$	0.215	0.129	−0.051 to 0.460	–	–	–
	$\sigma_{d_{s}}^{2}/\sigma_{d_{i}}^{2}$	0.164	0.045	0.083 to 0.260	–	–	–
	$\sigma_{m_{s}}^{2}/\sigma_{m_{i}}^{2}$	0.170	0.044	0.099 to 0.267	–	–	–
W210	$\sigma_{d}^{2}$	286.698	30.428	230.108 to 349.021	98.198	5.570	87.560 to 109.307
	$\sigma_{m}^{2}$	210.659	30.545	153.761 to 273.906	38.787	3.560	32.170 to 46.338
	$\sigma_{pm}^{2}$	120.340	12.948	95.290 to 146.20	57.233	1.877	53.550 to 60.990
	$c^{2}$	0.163	0.018	0.127 to 0.200	0.150	0.005	0.140 to 0.160
	$\sigma_{e}^{2}$	232.043	11.000	210.500 to 253.750	189.93	2.575	184.900 to 194.800
	$r_{d_{is}}$	−0.063	0.087	−0.235 to 0.103	0.607	0.045	0.526 to 0.708
	$r_{m_{is}}$	0.198	0.128	−0.064 to 0.441	0.392	0.085	0.219 to 0.546
	$\sigma_{d_{s}}^{2}/\sigma_{d_{i}}^{2}$	0.190	0.045	0.108 to 0.287	0.112	0.021	0.073 to 0.157
	$\sigma_{m_{s}}^{2}/\sigma_{m_{i}}^{2}$	0.159	0.038	0.095 to 0.243	0.120	0.026	0.072 to 0.174
SC	$\sigma_{d}^{2}$	–	–	–	38.837	1.485	35.978 to 41.789
	$\sigma_{e}^{2}$	–	–	–	3.612	0.911	3.434 to 3.790
	$r_{d_{is}}$	–	–	–	0.313	0.057	0.201 to 0.419
	$\sigma_{d_{s}}^{2}/\sigma_{d_{i}}^{2}$	–	–	–	0.029	0.006	0.019 to 0.042
DFC	$\sigma_{d}^{2}$	62.612	15.708	35.954 to 96.952	16.832	3.742	10.873 to 25.451
	$\sigma_{e}^{2}$	222.314	9.133	204.400 to 240.700	239.606	3.124	233.100 to 245.400
	$r_{d_{is}}$	−0.073	0.188	−0.422 to 0.311	0.550	0.130	0.286 to 0.754
	$\sigma_{d_{s}}^{2}/\sigma_{d_{i}}^{2}$	0.651	0.230	0.309 to 1.195	0.484	0.210	0.181 to 0.990

**Table 3:** Posterior means of genetic correlations (standard deviation) for direct and maternal effects of body weight at around 120 (W120) and 210 (W210) days of age, scrotal circumference (SC), and days to first calving (DFC) of Nelore cattle from an experimental (EXP) herd and from a company (COM) according to the gestational environment (GE, in standard deviations).

	Experimental Data (EXP)			
	W120 (direct effects: below diagonal, maternal above)			
GE	−2.5	−1.0	+1.0	+2.5
−2.5	1	0.901 (0.03)	0.622 (0.08)	0.343 (0.11)
−1.0	0.909 (0.02)	1	0.899 (0.03)	0.713 (0.06)
+1.0	0.639 (0.08)	0.900 (0.03)	1	0.947 (0.01)
+2.5	0.357 (0.12)	0.710 (0.07)	0.946 (0.01)	1
	W210 (direct effects: below diagonal, maternal above)			
−2.5	1	0.908 (0.02)	0.645 (0.07)	0.371 (0.10)
−1.0	0.929 (0.01)	1	0.904 (0.02)	0.722 (0.06)
+1.0	0.646 (0.07)	0.882 (0.03)	1	0.948 (0.01)
+2.5	0.284 (0.11)	0.617 (0.07)	0.914 (0.02)	1
	DFC (above)			
−2.5	1	0.885 (0.04)	0.241 (0.17)	−0.294 (0.16)
−1.0	–	1	0.657 (0.10)	0.172 (0.17)
+1.0	–	–	1	0.849 (0.05)
+2.5	–	–	–	1
	Company data (COM)			
	W210 (direct effects: below diagonal, maternal above)			
−2.5	1	0.957 (0.01)	0.869 (0.03)	0.640 (0.06)
−1.0	0.959 (0.01)	1	0.975 (0.01)	0.834 (0.03)
+1.0	0.885 (0.03)	0.889 (0.02)	1	0.936 (0.01)
+2.5	0.722 (0.06)	0.885 (0.03)	0.961 (0.01)	1
	SC (below), DFC (above)			
−2.5	1	0.818 (0.07)	0.502 (0.18)	0.102 (0.25)
−1.0	0.991 (0.00)	1	0.904 (0.05)	0.640 (0.14)
+1.0	0.969 (0.01)	0.994 (0.00)	1	0.904 (0.04)
+2.5	0.892 (0.02)	0.945 (0.01)	0.976 (0.01)	1

**Table 4:** Three main genomic regions that explained at least 0.5% of the genetic variance (var, %) for the intercept of reaction norms of direct and maternal effects of body weight at around 120 (W120) and 210 (W210) days of age and days to first calving (DFC) of Nelore cattle from an experimental herd.

Trait	BTA	Position (bp)	Var (%)	Candidate Genes	QTL
W120 (direct)	25	40,619,157–41,019,157	1.26	GNA12, AMZ1, BRAT1, bta-mir-11980, IQCE, TTYH3, LFNG, bta-mir-12029, GRIFIN, CHST12, bta-mir-12019, EIF3B, SNX8, NUDT1, MRM2, MAD1L1	Residual feed intake, conception rate, milking speed, average daily gain
	15	45,597,833–45,997,833	1.23	RBMXL2, NLRP14, ZNF214, ZNF215, OR2D3, OR2D2, OR10A4, OR10A5, U6, OR10A5L, OR10A5G, OR6A2, OR6B18, OR6B17, OR2D4	Milk-fat yield, body weight gain
	10	52,283,853–52,683,853	0.95	ALDH1A2, U6, POLR2M, MYZAP	Carcass weight, milk butyric acid content, milking speed, marbling score, m. paratuberculosis susceptibility, shear force, milk kappa-casein percentage, omega-6 to omega-3 fatty acid ratio, age at puberty
W120 (maternal)	7	21,907,628–22,307,628	0.99	IRF1, SLC22A5, SLC22A4, bta-mir-2457, PDLIM4, P4HA2, bta-mir-12040	Milking speed, m. paratuberculosis susceptibility, calving ease (maternal), daughter pregnancy rate, stillbirth (maternal), udder attachment, net merit, length of productive life, somatic cell score, stillbirth, udder depth
	2	54,729,312–55,129,312	0.73	-	milk palmitoleic acid content, inseminations per conception, bovine respiratory disease susceptibility
	8	52,728,463–53,128,463	0.64	PRUNE2, FOXB2	Residual feed intake, twinning, milk unglycosylated kappa-casein percentage, milk kappa-casein percentage, bovine tuberculosis susceptibility, milk protein yield
W210 (direct)	25	40,6191,57–41,019,157	0.94	GNA12, AMZ1, BRAT1, bta-mir-11980, IQCE, TTYH3, LFNG, bta-mir-12029, GRIFIN, CHST12, bta-mir-12019, EIF3B, SNX8, NUDT1, MRM2, MAD1L1	Residual feed intake, conception rate, milking speed, average daily gain
	21	14,794,265–15,194,265	0.71	SLCO3A1	Bovine tuberculosis susceptibility, age at first calving, kidney, pelvic, heart fat percentage, milk tridecylic acid content, somatic cell score
	15	45,597,833–45,997,833	0.64	RBMXL2, NLRP14, ZNF214, ZNF215, OR2D3, OR2D2, OR10A4, OR10A5, U6, OR10A5L, OR10A5G, OR6A2, OR6B18, OR6B17, OR2D4	Milk-fat yield, body weight gain
W210 (maternal)	8	52,728,463–53,128,463	0.94	PRUNE2, FOXB2	Residual feed intake, twinning, milk unglycosylated kappa-casein percentage, milk kappa-casein percentage, bovine tuberculosis susceptibility, milk protein yield
	11	94,730,926–95,130,926	0.75	DENND1A, LHX2	cheese protein recovery, number of embryos, milk beta-lactoglobulin percentage, anti-müllerian hormone level, non-return rate
	29	15,282,330–15,682,330	0.68	-	milk kappa-casein percentage, milk glycosylated kappa-casein percentage, milk unglycosylated kappa-casein percentage, somatic cell score
DFC	10	66,575,785–66,975,785	1.32	CDKN3, CNIH1	Milking speed, bovine tuberculosis susceptibility, milk protein yield, body depth, PTA type, udder attachment, udder height, rump width, somatic cell score, stature, strength, udder depth
	7	29,486,606–29,886,606	0.86	-	Clinical mastitis, body weight (birth), milk protein yield, body capacity, daughter pregnancy rate, stillbirth (maternal), udder attachment, length of productive life, somatic cell score, stillbirth, udder depth
	2	89,254,217–89,654,217	0.80	AOX4, AOX2, BZW1, CLK1, PPIL3, NIF3L1, ORC2, FAM126B, U6	Conception rate, intramuscular fat, milk-fat yield

**Table 5:** Three main genomic regions that explain at least 0.5% of the genetic variance (var, %) for the slope of reaction norms of direct and maternal effects of body weight at around 120 (W120) and 210 (W210) days of age and days to first calving (DFC) of Nelore cattle from an experimental herd.

Trait	BTA	Position (bp)	Var (%)	Genes	QTL
W120 (direct)	22	17,317,952–17,717,952	1.77	SRGAP3, RAD18	Milk tridecylic acid content, body weight (yearling), lean-meat yield, white spotting
	9	89,328,896–89,728,896	0.82	7SK, MYCT1, VIP	muscle magnesium content, muscle phosphorus content, teat placement—front, teat placement—rear, teat length, udder cleft
	26	40,536,474–40,936,474	0.74	PLPP4, WDR11	milk-fat yield, stature, milk c14 index, milk myristoleic acid content, milk yield, milk protein yield
W120 (maternal)	2	89,254,217–89,654,217	1.96	AOX4, AOX2, BZW1, CLK1, PPIL3, NIF3L1, ORC2, FAM126B, U6	Conception rate, intramuscular fat, milk-fat yield
	10	5,768,117–6,168,117	0.85	-	body weight (yearling), body weight gain, udder depth, conception rate
	23	22,140,708–22,540,708	0.81	MMUT, CENPQ, GLYATL3, C23H6orf141, U6, RHAG, CRISP2, CRISP3, 7SK, CRISP1	milk protein percentage, milk glycosylated kappa-casein percentage, milk iron content, length of productive life, daughter pregnancy rate, stillbirth (maternal), calving ease, somatic cell score
W210 (direct)	29	6,736,030–7,136,030	0.71	GRM5	Shear force
	7	36,882,466–37,282,466	0.68	-	Milk alpha-s1-casein percentage, milking speed, intramuscular fat
	15	54,575,598–54,975,598	0.63	RPS3, SNORD15, KLHL35, GDPD5, SERPINH1, MAP6, MOGAT2	Milk-fat yield, first service conception, inseminations per conception, 305-day milk yield, milk rennet coagulation time, bovine respiratory disease susceptibility, conception rate
W210 (maternal)	29	6,736,030–7,136,030	0.89	GRM5	Shear force
	5	50,640,891–51,040,891	0.69	PPM1H, MON2	Milk-fat yield, milk yield, inhibin level, insulin-like growth factor 1 level, intramuscular fat
	24	1,244,237–1,644,237	0.53	-	Body weight (yearling)
DFC	11	76,492,925–76,892,925	1.09	-	Body weight (yearling), milk alpha-lactalbumin percentage, lean-meat yield
	2	111,967,207–112,367,207	1.04	WDFY1, U6, MRPL44, SERPINE2	Metabolic body weight, fecal larva count, first service conception, heat tolerance, fertilization rate, conception rate, milk protein percentage, milk-fat yield
	1	149,053,882–149,453,882	0.84	HLCS, RIPPLY3, U6, PIGP, TTC3	Conception rate, somatic cell score, teat length, length of productive life

## Data Availability

The original data used in this research are available by contacting the corresponding author upon request.
